# Obesity phenotypes in urban and rural Cameroonians: a cross-sectional study

**DOI:** 10.1186/s13098-015-0016-5

**Published:** 2015-03-18

**Authors:** Vivian N Mbanya, Justin B Echouffo-Tcheugui, Hussain Akhtar, Jean-Claude Mbanya, Andre P Kengne

**Affiliations:** Department of Community Medicine, Section of International Health, University of Oslo, Oslo, Norway; Hubert Department of Global Health, Rollins School of Public Health, Emory University, Atlanta, GA 30322 USA; Department of Medicine, MedStar Health System, Baltimore, MD 21239 USA; Health of Populations in Transition (HoPiT) Research Group, Faculty of Medicine and Biomedical Sciences, The University of Yaoundé 1, Yaoundé, Cameroon; Non-Communicable Diseases Research Unit, South African Medical Research Council, Cape Town, Western Cape 7505 South Africa; Department of Medicine, Groote Schuur Hospital, University of Cape Town, Cape Town, South Africa; The George Institute for Global Health, The University of Sydney, Sydney, NSW 2050 Australia

## Abstract

**Background:**

Despite the increasing prevalence of diabetes and other health consequences of obesity, little is known on the metabolic profile across categories of body mass index (BMI) among African populations. We therefore assessed the prevalence and distribution of body size phenotypes among urban and rural Cameroonians.

**Methods:**

Adults (n = 1628; 41% rural dwellers) aged 24–74 years in 1994 provided data on BMI and metabolic health, defined on the basis of elevated levels of blood pressure (BP); triglycerides, fasting plasma glucose (FPG), and insulin resistance as assessed with homeostasis model assessment (HOMA). Cross-classification of BMI categories and metabolic status (healthy/unhealthy) created six groups. Metabolic measures include elevated blood pressure; elevated triglycerides (≥150 mg/dL or 1.69mmo/L), elevated fasting plasma glucose (≥100 mg/dl or 5.6 mmol/L or documented use of antidiabetic medications), and elevated homeostasis model assessment of insulin resistance value (HOMA-IR > 90^th^ percentile).

**Results:**

A total of 25.2% of participants were overweight yet metabolically healthy (<1 abnormality) and 10.1% were obese yet metabolically healthy, whereas 1.4% were normal weight but metabolically abnormal (≥2 abnormalities). Proportion of rural dwellers with abnormal metabolic phenotype across normal-weight, overweight, obese categories were 2.9%, 0.8% and 0.3%, respectively; and 0 .3%, 2.2% and 2.6% among urban dwellers. Metabolically abnormal participants increased linearly across BMI categories (p < 0.001). BMI categories and metabolic status interacted to affect age, gender, BMI, FPG, triglycerides, and BP status distributions (all p < 0.04). Metabolic status and residence (rural vs. urban) interacted to influence the distribution across BMI categories of diastolic BP, BMI, waist circumference, fasting and 2-hour glucose, triglycerides, HOMA-IR, and prevalent diabetes (all p < 0.005), with differential occurrence of BMI categories and metabolic status among urban and rural participants.

**Conclusions:**

Metabolic healthy obesity and obesity with a favorable cardiometabolic profile are not uncommon among Cameroonians, including among rural dwellers; but the latter group tended to have a better profile.

## Background

Obesity is a major precursor of type 2 diabetes mellitus and several cardiovascular disease (CVD) risk factors including hypertension, dyslipidemia and pro-inflammatory states. There has been an increasing recognition that the disease risks associated with obesity may not be uniform [1]. Indeed variations in metabolic and CVD risk factors have been observed among individuals of similar body mass index (BMI) [2]. Furthermore studies suggest than an individual’s CVD risk may depend jointly on their body size and metabolic profile [3]. Consequently, there have been several investigations on body size phenotypes, with a recognized phenotype being the metabolically healthy but obese individual, also referred to as “uncomplicated” obesity [4], which applies to people who although obese appear to be relatively resistant to the development of the adiposity-associated metabolic abnormalities that would increase CVD risk [3]. Another body size phenotype includes individuals with normal weight, who express metabolic abnormalities often associated with being overweight and obese [2].

Despite the increasing prevalence of obesity in Africa population [5], assessment of the aforementioned body size phenotypes and their implications for disease risk and prognostic have mainly been conducted in Western populations [6]. Furthermore, few extant studies have examined the frequencies of various phenotypes by ethnicity, with suggestion of higher prevalence of metabolically healthy obesity among Asians populations [4]. Little is known regarding their prevalence and pattern in the African context, especially as there may be a rural –urban divide that may explain the distributions of body size phenotypes, and accordingly some of the observed urban-rural differentials in the distribution of major CVD risk factors in Africa. Therefore, we investigated a representative sample of adult Cameroonians, in order to assess: (i) the prevalence of body size phenotypes (normal weight with and without cardiometabolic abnormalities, overweight with and without cardiometabolic abnormalities, and obese with and without cardiometabolic abnormalities), (ii) the characteristics of expressing cardiometabolic abnormalities if normal weight, and (iii) the characteristics of appropriate metabolically health (no cardiometabolic risk factor clustering) if overweight or obese.

## Methods

### Study participants

The study was a cross-sectional survey conducted in 1994 among Cameroonians, in three villages of the Evodoula (rural) and the Cité Verte (urban) health areas. All inhabitants, aged between 24 and 74 years and who had been residents at the sites for at least 1 year before the surveys were recruited through a simple probability sampling design. The response rates were 95 and 91% in the rural and urban sites, respectively. Of the 1986 participants (786 rural and 1160 urban subjects), complete data on variables of interest were available for 1628 (669 rural and 959 urban subjects) participants, thus constituting the final sample. The study procedures have been described in details previously [7]. All individuals gave informed consent to participate. The studies were approved by relevant ethics committees in Cameroon, and conformed to the principles outlined in the declaration of Helsinki.

### Measurement of demographic, health and physical factors

Age, sex, educational level, smoking status, alcohol intake, physical activity, history of hypertension or diabetes, as well as the use of antihypertensive, lipid-lowering, and antidiabetic medications were assessed by self-report. Smoking status was categorized as non-smoker (had never smoked), ex-smoker (had stopped smoking for at least one year) and smokers (current smokers). Alcohol consumption was based on the intake of alcoholic beverages during the last year, with two categories heavy drinkers vs. non- heavy drinkers. The type of last educational institution attended was used, giving two categories: lower (<7 years of education), and higher (including secondary (7 to 14 years) and university (more than 14 years) levels]. The Sub-Saharan Africa Activity Questionnaire (SSAAQ) [8] was used to assess the leisure time physical activity during the past month. Frequency and duration were computed for each reported activity, and the energy expenditure was calculated using Ainsworth et al.’s compendium [9]. Energy expenditure related to leisure time physical activity was calculated by multiplying the ratio of the exercise to resting metabolic rate (MET, Metabolic equivalent) score by the number of hours spent in each activity. From the total MET of each subject, we categorized physical activity into strenuous vs. non-strenuous.

Height (to the nearest centimetre) and weight (to the nearest kilogram) were measured using standard methods and body mass index (BMI) calculated for all subjects. They were classified as underweight (BMI <18.5 kg/m^2^), normal weight (BMI ≥18.5 and BMI <25 kg/m^2^), overweight (BMI ≥25 kg/m^2^ and BMI <30 kg/m^2^) and obese (BMI ≥30 kg/m^2^). Waist circumference was measured to the nearest 0.1 cm at the level of the iliac crest at the end of normal respiration [10,11]. Waist to hip ratio (WHR) was the waist circumference divided by the hip circumference.

### Measurement of cardiometabolic components

The four metabolic components measured include elevated blood pressure; elevated levels of triglycerides (≥150 mg/dL or 1.69 mmo/L), fasting plasma glucose (≥100 mg/dl or 5.6 mmol/L or documented use of antidiabetic medications), and elevated homeostasis model assessment of insulin resistance value (HOMA-IR > 90^th^ percentile). Seated (after at least 30 minutes) diastolic (DBP) and systolic (SBP) blood pressures were measured using a mercury sphygmomanometer with three recordings on the right arm using a standard mercury sphygmomanometer and appropriate cuff sizes. The average of the second and third measures was used to define hypertension. Elevated BP was defined as: SBP ≥ 140 mmHg, and/or DBP ≥ 90 mmHg, or use of blood pressure lowering drugs. The OGTT was performed according to the World Health Organization (WHO) protocol [12], with the subjects ingesting glucose in 300 ml of chilled water corresponding to 75-g of anhydrate glucose (Plantecam/Medicam, Mutengene, Cameroon). Venous blood samples were collected after an overnight fast of at least 12 hours for the determination of plasma glucose, insulin, and triglycerides levels. Samples for insulin determination were collected on ice, centrifuged immediately, separated and stored at −70°C until assayed. Fasting plasma glucose (FPG) was determined by the glucose-oxidase method using a spectrophotometer with external quality control on every 4^th^ sample by a Cobas bio hexokinase fluorometric method. Plasma insulin was assayed at the Welcome Laboratories, University of Newcastle Upon Tyne, by ELISA method using DAKO kits (Hersteller, UK). Triglycerides were measured by enzymatic colorimetric methods in the same laboratory. Homeostasis assessment model for insulin resistance (HOMA-IR) was used to evaluate insulin resistance using the following formula: Fasting plasma glucose (mmol/l) × Fasting plasma insulin (μU/ml)/22.5.

### Body size phenotype definitions

No standardized definition of body size phenotypes exists. Hence, for the present analyses, four metabolic abnormalities were considered (elevated blood pressure; elevated triglyceride and glucose levels; insulin resistance). Body size phenotypes were defined based on the combined consideration of BMI category (normal weight, overweight, and obesity) and having 0 to 1 (metabolically healthy) or 2 or more (metabolically abnormal) cardiometabolic abnormalities. The categories were normal weight and metabolically healthy (BMI < 25.0 kg/m^2^ and < 2 cardiometabolic abnormalities), normal weight and metabolically abnormal (BMI < 25.0 kg/m^2^ and ≥ 2 cardiometabolic abnormalities), overweight, metabolically healthy (BMI 25.0-29.9 kg/m^2^ and <2 cardiometabolic abnormalities), overweight and metabolically abnormal (BMI 25.0-29.9 kg/m^2^ and ≥ 2 cardiometabolic abnormalities), obese and metabolically healthy (BMI ≥ 30.0 kg/m^2^ and <2 cardiometabolic abnormalities), and obese and metabolically abnormal (BMI ≥30.0 kg/m^2^ and ≥2 cardiometabolic abnormalities).

### Statistical analysis

Participant characteristics are presented as means (standard deviation) or median (interquartile range) or percentages, by site of residence (urban vs. rural) and by body size phenotype. Pairwise comparisons within BMI category, for metabolically healthy and metabolically abnormal individuals were tested using chi square tests, and t-tests and non-parametric equivalents as appropriate. The linear trends across BMI categories, separately for metabolically healthy and metabolically abnormal participants were tested via Cochran-Artmitage trend tests, and Brown-Forsythe Levene procedures. Two-ways interactions between BMI categories and metabolic status, and between setting (rural or urban) and metabolic status were tested as well as the 3-ways interaction setting (rural or urban), BMI categories, and metabolic status. A two-sided p-value < 0.05 was used to define statistical significance. All analyses were conducted using the Statistical Package for Social Sciences (SSPS Inc., Chicago, IL) version 17.0 software.

## Results

### Participant’s characteristics

As shown in Table 1, compared to urban dwellers those living in the rural settings were more likely to be older (45.9 vs. 37.6 years, p < 0.001), of lower socio-economic status (93.2% vs. 45.6%, p < 0.001) and more physically active (57.2 vs. 37.8, p < 0.001); but less likely to be educated (secondary education or more: 20.2% vs. 89.8%, p < 0.001), have higher diastolic blood pressure (72 vs. 77 mmHg, p < 0.001), be on antihypertensive drugs or have hypertension (5.3% vs. 9.9%, p = 0.001), be obese (as estimated by BMI and WHR; both p < 0.001) or glucose intolerant or insulin resistant (both p < 0.001).

**Table 1:** Baseline characteristics of participants (urban vs. rural)*

**Variables**	**Overall**	**Rural**	**Urban**	**p-value**
N	1628	669	959	
Men, n (%)	691 (42.4)	272 (40.7)	419 (43.7)	0.223
Age, years (SD)	41.0 (11.7)	45.9 (13.3)	37.6 (9.1)	<0.001
Systolic blood pressure, mmHg (SD)	117 (18)	117 (20)	117 (17)	0.991
Diastolic blood pressure, mmHg (SD)	75 (13)	72 (12)	77 (13)	<0.001
Body mass index, kg/m^2^ (SD)	24.5 (4.4)	22.1 (3.1)	26.1 (4.5)	<0.001
Waist circumference, cm (SD)	82 (9)	82 (9)	82 (10)	0.050
Waist-to-hip ratio (SD)	0.85 (0.12)	0.81 (0.07)	0.91 (0.15)	<0.001
Fasting blood glucose, mmol/L (SD)	4.2 (1.5)	4.2 (1.6)	4.2 (1.4)	0.261
2h glucose, mmol/L (SD)	5.1 (2.0)	5.0 (1.7)	5.3 (2.3)	0.002
Triglycerides, mmol/L (SD)	0.53 (0.31)	0.52 (0.26)	0.54 (0.34)	0.522
Total cholesterol, mmol/L (SD)	3.1 (0.9)	3.6 (0.7)	3.5 (0.9)	<0.001
Fasting insulin, U/L [25th-75th percentiles]	3.7 [2.2-6.0]	2.4 [1.4-3.7]	4.8 [3.3-7.1]	<0.001
Fasting c-peptide, U/L [25th-75th percentiles]	0.28 [0.19-0.40]	0.23 [0.16-0.34]	0.31 [0.21-0.43]	<0.001
Total energy, METs (SD)	3726 (1516)	4087 (1704)	3491 (1316)	<0.001
HOMA-IR, Units [25th-75th percentiles]	0.67 [0.39-1.12]	0.42 [0.26-0.68]	0.88 [0.57-1.35]	<0.001
Education (secondary or more), n (%)	979 (62.4)	125 (20.2)	854 (89.8)	<0.001
Low socio-economic status, n (%)	1057 (65.1)	620 (93.2)	437 (45.6)	<0.001
Heavy drinker, n (%)	208 (12.8)	150 (22.6)	58 (6.1)	<0.001
Smoking, n (%)				<0.001
Never	1366 (84.1)	579 (86.9)	787 (82.2)	
Ex-smoker	92 (5.7)	14 (2.1)	78 (8.1)	
Current smokers	166 (10.2)	73 (11.0)	93 (9.7)	
Strenuous physical activity, n (%)	747 (45.9)	383 (57.2)	363 (37.8)	<0.001
Any Diabetes, n (%)	65 (4.0)	33 (5.0)	32 (3.3)	0.102
Any hypertension, n (%)	130 (8.0)	35 (5.3)	95 (9.9)	0.001

*Values are means and standard deviation (SD), median and 25th-75th percentiles or count and percentages.

### Prevalence of body size

**Figure 1:**
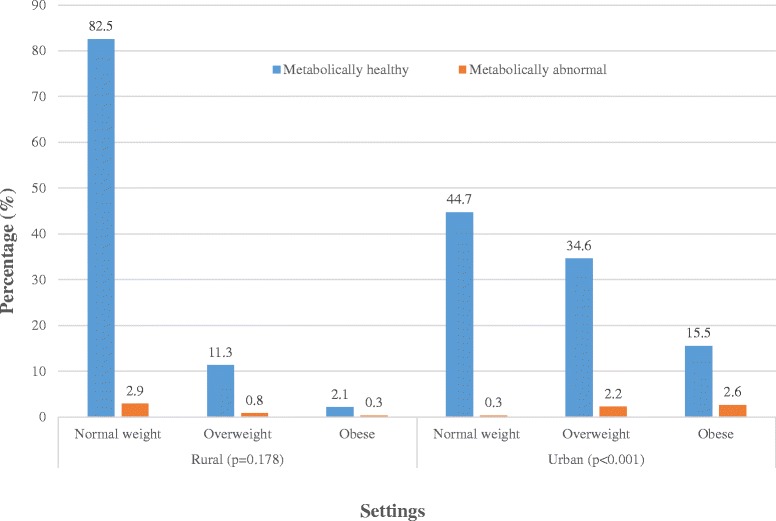
**Distribution of obesity phenotypes in urban and rural Cameroonians.**

Among the investigated adult Cameroonians (aged 24 to 74 years), 25.2% were overweight yet metabolically healthy (0 or 1 metabolic abnormality) and 10.1% were obese yet metabolically healthy, whereas 1.4% were normal weight but metabolically abnormal (≥2 metabolic abnormalities). Among rural dwellers, the proportion of abnormal metabolic phenotype across normal-weight, overweight, obese categories were 2.9%, 0.8%, 0.3%, respectively; the corresponding values for urban dwellers were 0 .3%, 2.2% and 2.6% (Figure 1). The proportion of metabolically abnormal participants increased significantly across BMI categories overall in a linear fashion (both p < 0.001), primarily driven by significant (p < 0.001) and linear increase (p < 0.001 for linear trend) in urban participants and linear decrease (p = 0.036) among rural participants, with significant interaction by setting (p < 0.001).

### Characteristics of metabolic profiles within BMI categories

Within BMI categories, metabolically abnormal participants were older (all p ≤ 0.024), and as expected had higher SBP and DBP (all p < 0.001), fasting and 2-hour post load glucose (p < 0.001), HOMA-IR (all p < 0.024) and high prevalence of hypertension or diabetes (both p < 0.001). They also tended to have higher waist circumference (all p ≤ 0.049), comprise more women (all p ≤ 0.073) and to have higher C-peptide levels (among overweight and obese only, both p ≤ 0.001). Furthermore, in the obese sub-group BMI tended to be higher among metabolically abnormal participants (34.3 vs. 33.0, p = 0.066), while in the normal weight subgroup metabolically abnormal were less likely to be educated (27.8% vs. 53.8%, p = 0.025) and more often heavy drinkers (31.8% vs. 14.7%, p = 0.036), Table 2.

**Table 2:** **Pairwise comparisons of metabolically healthy and metabolically abnormal participants with body mass index categories**

**Characteristics**	**Normal-weight**			**Overweight**			**Obese**			**P trend across BMI categories**			**P interaction**		
**Metabolic status**	**Healthy**	**Abnormal**	**p**	**Healthy**	**Abnormal**	**p**	**Healthy**	**Abnormal**	**p**	**Overall**	**Healthy**	**Abnormal**	B*M	M*S	B*M*s
Prevalence, n (%)	968 (60.0)	22 (1.4)		406 (25.2)	26 (1.6)		163 (10.1)	27 (1.7)		<0.001	-	-	<0.001		
Men	426 (54.3)	8 (36.4)	0.073	161 (39.7)	17 (65.4)	0.009	39 (23.9)	10 (37.0)	0.116	<0.001	<0.001	0.054	0.002	0.541	0.244
Age (years)	40.9 (12.9)	54.2 (10.8)	<0.001	39.4 (9.1)	43.9 (10.2)	0.016	41.7 (9.3)	46.1 (9.4)	0.024	0.922	0.641	0.010	0.014	0.650	0.004
Systolic blood pressure	114 (16)	146 (33)	<0.001	118 (18)	141 (17)	<0.001	122 (17)	143 (23)	<0.001	<0.001	<0.001	0.643	0.082	0.421	0.049
Diastolic blood pressure	72 (11)	83 (15)	<0.001	77 (13)	93 (17)	<0.001	80 (12)	94 (17)	<0.001	<0.001	<0.001	0.032	0.477	0.004	0.060
Body mass index	21.7 (2.0)	21.5 (2.3)	0.729	27.0 (1.3)	27.2 (1.3)	0.525	33.0 (3.1)	34.3 (4.8)	0.066	<0.001	<0.001	<0.001	0.044	<0.001	0.021
Waist circumference	78 (7)	81 (6)	0.049	86 (7)	90 (6)	0.009	95 (9)	100 (9)	0.004	<0.001	<0.001	<0.001	0.513	<0.001	<0.001
Waist-hip-ratio	0.86 (0.12)	0.89 (0.04)	0.166	0.83 (0.09)	0.87 (0.05)	0.039	0.83 (0.10)	0.86 (0.09)	0.200	<0.001	<0.001	0.051	0.934	0.006	0.004
Fasting blood glucose	4.0 (0.6)	7.6 (8.3)	<0.001	4.2 (0.8)	6.3 (4.9)	<0.001	4.1 (0.6)	5.7 (4.6)	<0.001	0.008	0.008	0.304	<0.001	0.003	<0.001
2h glucose	4.9 (1.1)	12.4 (11.1)	<0.001	4.9 (1.0)	9.6 (6.7)	<0.001	5.0 (1.0)	7.9 (2.8)	<0.001	0.006	0.134	0.048	<0.001	<0.001	<0.001
Triglycerides	0.51 (0.27)	0.55 (0.18)	0.408	0.54 (0.32)	1.06 (1.04)	<0.001	0.58 (0.23)	0.72 (0.25)	0.004	<0.001	0.001	0.430	<0.001	0.002	<0.001
Total cholesterol	2.9 (0.8)	2.9 (0.9)	0.885	3.5 (0.9)	3.3 (0.8)	0.428	3.8 (0.9)	3.9 (0.7)	0.501	<0.001	<0.001	<0.001	0.568	0.407	0.109
Total energy	3857 (1579)	3704 (1449)	0.652	3574 (1408)	3380 (1029)	0.492	3435 (1396)	3438 (1185)	0.993	<0.001	<0.001	0.473	0.895	0.431	0.505
Fasting insulin	2.9 [1.8-4.6]	2.9 [2.1-11.9]	0.915	4.8 [3.3-6.7]	7.2 [6.0-13.9]	<0.001	.0 [4.0-11.4]	15.2 [9.6-18.0]	<0.001	<0.001	<0.001	<0.001	0.161	0.057	0.439
Fasting c-peptide	0.24 [0.17-0.34]	0.29 [0.22-0.68]	0.320	0.32 [0.22-0.44]	0.44 [0.32-0.62	0.001	0.37 [0.27-0.48]	0.71 [0.56-0.92]	<0.001	<0.001	<0.001	0.001	0.012	0.027	0.194
HOMA_IR	0.52 [0.32-0.83]	1.22 [0.32-3.32]	0.024	0.88 [0.59-1.30]	2.28 [1.28-2.71]	<0.001	1.11 [0.75-1.62]	3.12 [2.48-3.90]	<0.001	<0.001	<0.001	0.003	0.118	0.027	0.222
Education (secondary or more)	497 (53.8)	5 (27.8)	0.025	301 (75.1)	21 (80.8)	0.349	128 (80.0)	22 (84.6)	0.403	<0.001	<0.001	<0.001	0.090	0.262	0.423
Low socio-economic status	723 (74.9)	19 (86.4)	0.164	208 (51.4)	7 (26.9)	0.013	77 (47.2)	11 (40.7)	0.339	<0.001	<0.001	0.003	0.063	0.869	<0.001
Heavy drinker	141 (14.7)	7 (31.8)	0.036	40 (9.9)	2 (7.7)	0.525	11 (6.7)	3 (11.1)	0.318	<0.001	0.001	0.062	0.354	0.805	0.142
Smoking			0.556			0.632			0.036	0.520	0.197	0.314	0.200	0.279	0.439
Never	816 (84.6)	17 (77.3)		337 (83.2)	23 (88.5)		142 (87.1)	18 (66.7)							
Ex-smoker	40 (4.1)	2 (9.1)		35 (8.6)	1 (3.8)		10 (6.1)	3 (11.1)							
Current smokers	109 (11.3)	3 (13.6)		33 (8.1)	2 (7.7)		11 (6.7)	6 (22.2)							
Strenuous physical activity	475 (49.1)	21 (54.5)	0.385	168 (41.4)	9 (34.6)	0.321	65 (39.9)	11 (40.7)	0.546	0.002	0.003	0.367	0.697	0.827	0.678
Any Diabetes	19 (2.0)	13 (68.4)	<0.001	4 (1.0)	13 (50.0)	<0.001	0	15 (55.6)	<0.001	0.007	0.032	0.445	0.996	0.041	0.020
Any hypertension	34 (3.5)	10 (45.5)	<0.001	36 (8.9)	11 (42.3)	<0.001	26 (16.0)	11 (40.7)	0.005	<0.001	<0..001	0.744	0.015	0.378	0.036

Values are count (percentages, %), mean (standard deviation, SD) or median [25^th^ – 75^th^ percentiles]. B*M, interaction term of body mass index categories and metabolic status; M*S, interaction term of metabolic status and setting (rural vs. urban); B*M*S, 3-ways interaction term of body mass index categories, metabolic status and setting. Units of measurements and other conventions are as per Table 1.

### Characteristics by metabolic profile across BMI categories

The proportion of men decreased from 54.3% in the normal-weight healthy subjects to 23.9% in the obese healthy group (p < 0.001 for linearity), while it was approximately the same among normal-weight and obese metabolically abnormal subjects (36.4% vs. 37.0%), but higher in the overweight metabolically abnormal subjects (65.4%) with non-significant linear trend (p = 0.054). Age decreased linearly across BMI categories among metabolically abnormal subjects (p = 0.01), but not among the healthy subjects (p = 0. 641). SBP and DBP, BMI, waist circumference, total cholesterol, fasting insulin, fasting C-peptide, HOMA-IR and education level increased in linear fashions (all p < 0.032) while socio-economic status (all p < 0.003) linearly decreased across BMI categories in both metabolically health and abnormal participants (Table 2). Hypertension rate (p < 0.001), diabetes frequency (p = 0.032), heavy drinking (p = 0.001), total energy intake (p < 0.001) and waist-hip-ratio (p < 0.001) linearly decreased across BMI categories among metabolically normal subjects, while the pattern was always non-significant among metabolically abnormal subjects (all p > 0.05 for linear trend), Table 2.

### Interactions by BMI, metabolic status and study setting (rural or urban)

There was significant interaction between BMI categories and metabolic status in the distribution of gender (p = 0.002), age (p = 0.014), BMI (p = 0.044), fasting and 2 h post load glucose (both p < 0.001), triglycerides (p < 0.001), fasting C-peptide (p = 0.012) and hypertension (p = 0.015). Furthermore, significant interactions were observed between metabolic status and setting (rural vs. urban) in the distribution across BMI categories of diastolic blood pressure (p = 0.004), BMI and waist circumference (both p < 0.001), waist-to-hip ratio (p = 0.006), fasting 2-hour post load glucose (both p < 0.003), triglycerides (p = 0.002), fasting c-peptide and HOMA-IR (both p = 0.027), and prevalent diabetes (p = 0.041), Table 2. There was also evidence of significant 3-ways interaction setting*[BMI categories]*metabolic status in the distribution of many characteristics as shown in Table 2, clearly indicating that most of the variations observed across BMI categories and metabolic status were occurring in differential ways among urban and rural participants.

## Discussion

Our study shows that a considerable proportion of overweight or obese Cameroonian adults are metabolically healthy, whereas a sizable proportion of normal-weight adults, particularly in rural settings express a clustering of cardiometabolic abnormalities. Up to 85.8% of obese Cameroonians possess a healthy profile, more so among those living in rural areas than in urban areas, in terms of the standard cardiometabolic risk factors. In contrast, 2.1% of normal-weight people exhibit clustering of cardiometabolic abnormalities (i.e., ≥2 cardiometabolic abnormalities). This study also found significant differences in the distribution of many characteristics across obesity phenotypes, with strong indications that these differences were occurring in differential ways between urban and rural participants.

Our study is among the firsts to examine the concept of metabolically healthy obesity in African populations, and is relevant to the ongoing debate on the usefulness of this concept. The prevalence of body size phenotypes among African populations including the urban rural differences, have seldom been investigated in studies [6]. Compared to studies conducted in other continents, and despite differences in the definitions of “metabolically healthy” that have been used, the prevalence of metabolically healthy obese individuals is similar between the present and previous studies, which found a prevalence of 6 to 75% [6], depending on the definition used, and higher in population of Asia descend than in Caucasian. People of African descent have seldom been included in existing studies. The prevalence of individuals who are normal weight yet have metabolic abnormalities has been less studied [13].

Interestingly, distributions of several characteristics across body size phenotype were affected by place of residence. Indeed, variations in BMI and metabolic status were function of the place of residence, with rural status tending to confer a better metabolic profile. This observation will need further confirmation on larger samples. The reasons for this interesting observation remain unclear. However, this suggests that BMI may not be the most appropriate accurate measure of the adverse effects of excess adiposity [14]. On may speculate that these differences may be related to lifestyle factors, namely diet and physical activity. Hence the exploration of lifestyle characteristics may help disentangle these issues. Also, contextual or environmental effects may play a role in the determining the extent of cardiometabolic health among obese people. If this were to be the case, it would call for tailoring of preventive strategies depending on the setting.

Our study has limitations that merit consideration. Our data was not representative of the entire Cameroonian population. Furthermore, our data dates back to the year 1994, and the demographic and nutrition transition may have led to different patterns of body size phenotypes in the current Cameroonian population, especially as some of the risk factors change over time [7,11]. However, this time lag does not invalidate the relevance of our data; although the contemporary proportions of people in various phenotype categories may vary, it is highly likely that over time the main change would be that more people would have become obese especially in urban area. Body size phenotype definitions have not been standardized, hence the prevalence estimates are subject to alteration depending on the number of metabolic abnormalities considered and the specific cut points of those abnormalities. In addition, BMI as a measure of obesity has limitations because it cannot distinguish between fat tissue and lean tissue. Whether this limitation is pertinent for African populations remains to be determined as the amount of body fat per given BMI value in these populations compared to other populations (e.g.; Western populations) remains to be clarified [15-19]. A similar limitation exists for waist circumference, as studies on African populations have seldom examined the relation of abdominal visceral to waist circumference [20], and its relation to outcomes. Another limitation was the cross-sectional design of our study, which precludes appropriate investigation and causal attribution.

Despite the aforementioned limitations, our study had a number of strengths. To our knowledge it is the first of its kind on African populations on body size phenotypes, and including a sizeable number of Cameroonians with investigation of urban vs. rural contrast, with robust measures abnormal metabolism including glucose intolerance through OGTT and insulin resistance.

## Conclusions

In conclusion, the present data suggest that a substantial proportion of individuals with normal-weight display cardiometabolic abnormality clustering, as well as an important fraction of obese individuals who are metabolically healthy. Additional and more representative, larger and longitudinal studies, including behavioural, hormonal or biochemical, and genetic factors, as well as relevant cardiovascular outcomes among population of African descent, which would help to designing and refining obesity intervention targets and improving screening tools.
